# Psychosocial Interventions to Improve Wellbeing in Teenage and Young Adult Post‐Treatment Survivors of Childhood Cancer: A Systematic Review

**DOI:** 10.1002/pon.70081

**Published:** 2025-02-07

**Authors:** Nicola O'Donnell, Leila Ellis, Jessica E. Morgan, Pernille Axél Gregersen, Victoria Willard, Debra Howell, Bob Phillips

**Affiliations:** ^1^ Paediatric Palliative Care Group University of York York UK; ^2^ Population Health Science Bristol Medical School University of Bristol Bristol UK; ^3^ University Hospitals Bristol and Weston NHS Foundation Trust Bristol UK; ^4^ Centre for Reviews and Dissemination University of York York UK; ^5^ Department of Clinical Genetics Aarhus University Hospital Aarhus Denmark; ^6^ Department of Psychology St. Jude Children's Research Hospital Memphis Tennessee USA; ^7^ The Hull York Medical School University of York Hull UK

**Keywords:** cancer, interventions, oncology, paediatrics, psychosocial, systematic review

## Abstract

**Objective:**

This systematic review explores psychosocial interventions aimed at improving the well‐being of teenage and young adult (TYA) survivors of childhood cancer. It focuses on post‐treatment survivors aged 13–39 years, examining the types of interventions trialled, their efficacy in enhancing different facets of mental well‐being, and any potential negative impacts. The review was prospectively registered on PROSPERO and utilised randomised controlled trials (RCTs) to assess psychosocial interventions such as cognitive‐behavioural therapy, psychoeducation, peer support, and physical activity programmes.

**Methods:**

Fifteen studies involving 1109 participants were included, covering interventions across multiple modes of delivery; online, in‐person, and app‐based.

**Results:**

Interventions demonstrated varied effects on outcomes like quality of life, depression, anxiety, stress, mood, behaviour, coping skills, and social support. While some interventions, such as psychoeducation and physical activity programs, showed promising improvements in well‐being, others demonstrated limited or mixed results. No significant adverse effects were reported.

**Conclusions:**

The heterogeneity of interventions and outcome measures pose challenges for definitive conclusions, emphasising the need for future research with standardised measures, consistent sample sizes, and long‐term follow‐up to better assess the sustainability of intervention effects. Overall, the findings highlight the importance of tailored psychosocial support to address the unique needs of TYA cancer survivors during their survivorship journey.

## 1 Background

It has been documented that once treated for cancer and off treatment, individuals and their families can be left ‘in limbo’ [1]. This captures the survivorship journey towards normality and life without active treatment, but with an impact on well‐being, potential additions of fear of recurrence and social difficulties when reintegrating into 'normal life' [2]. Psychosocial interventions are used to reduce such difficulties but little is known about their feasibility and efficacy, particularly for childhood cancer survivors who are now teenagers and young adults (TYA) [3, 4]. TYA in cancer settings are situated in the crossover of healthcare systems aimed at either children or adults [5, 6]. For this reason, the psychological support needs of survivors of childhood cancer have been named as a James Lind Alliance (JLA) top 10 priority [7].The psychosocial impact of childhood cancer on this group can be vast, as many experience interrupted development, impacting cognitive and social outcomes [8]. It follows that tailored psychosocial care and interventions should be offered to respond flexibly to the needs of individuals at this life stage [9].

Psychoeducation interventions involve therapeutically providing individuals with relevant and up‐to‐date information about their health to support them to live with and/or beyond a diagnosis. Interventions can also include peer discussion and social support, drawing upon the evidence‐base to offer problem‐solving and coping skills training [10]. This approach has been successfully utilised in oncology, anxiety, and trauma [11, 12, 13]. Psychoeducation can take many forms and has typically involved app‐ or website‐based interventions, group and individual workshops [14, 15, 16, 17, 18, 19, 20].

Existing reviews highlight many possible long‐term psychological consequences of childhood cancer, including depression, anxiety, behavioural difficulties, drug misuse and body image concerns. Mental wellness can be defined in several ways, with the World Health Organisation (WHO) referring to an individual realising their own ability, being able to cope with life stresses, and contributing to their work and community [21]. The Faculty of Public Health expands upon this to include the capacity to form positive relationships with others, experience contentment and joy, have confidence, and take responsibility for oneself [22].

To improve wellbeing, evidence‐based and effective psychosocial support is warranted and wanted [23, 24, 25]. The evaluation of such interventions is best conducted in randomised clinical trials (RCT) to estimate the benefit of such interventions, as well as considering feasibility, factors which prevent engagement, and the ‘cost versus benefit’ for the mental wellness of survivors [24, 25, 26].

This systematic review aimed to explore psychosocial interventions designed for TYA survivors of childhood cancer. Specifically, it aimed to answer the following questions:

- What types of psychosocial interventions have been trialled for TYA survivors of childhood cancer?
- Is there a psychosocial intervention that provides higher efficacy in improving survivors' mental well‐being?
- Do psychosocial interventions positively influence the well‐being and psychological health of TYA survivors, and are there any possible negative impacts or ‘adverse events’?

## 2 Methods

A protocol was registered prospectively on PROSPERO (CRD42023422933). This review is reported in line with the Preferred Reporting Items for Systematic Reviews and Meta‐Analyses (PRISMA) statement [27]. A patient and public involvement (PPI) group steered the review, providing insight into important reporting outcomes, identified research needs, and were involved in dissemination.

### 2.1 Searches

Searches were conducted for studies which evaluated any intervention which targeted 13–39‐year‐old TYA post‐treatment survivors of any type of cancer. This age range was determined to incorporate both the UK and United States of America definitions of ‘TYA’, as per National Cancer Institute [28], Cancer Research UK [29], and JLA [6]. Studies published from any year were searched for in the databases of MEDLINE ALL, PsycINFO, Scopus, the Cochrane Library, CINAHL (EBSCO), British Nursing Database, PsycARTICLES, and EMBASE. PROSPERO searches and clinical trial registries were also conducted to identify unpublished or ongoing reviews and studies on similar topics. Forward and backward citation searches were then performed. Owing to time and resource constraints, only articles written in English were included.

## 3 Selection Process

### 3.1 Study Selection

NOD conducted all database searches and imported these into Covidence reference management software [30]. LE replicated these searches independently to check for reliability. Any duplicate or irrelevant articles were identified, noted, and removed. NOD screened all remaining texts by title and abstract for possible inclusion. Reference lists of all relevant articles were also searched. LE independently screened a sample of 10% titles and abstracts. Of the 847 papers screened by both reviewers, there was 0.92 proportionate agreement, and Cohen's Kappa of 0.64 showing good levels of agreement. For papers where there were conflicts, consensus discussion revealed varied ‘maybe’ decisions, and agreement was reached. There was no need for any papers to be referred to a third reviewer.

All full texts were considered for eligibility by NOD and LE with both independently screening 100% of full texts. Any disagreements over inclusion were managed using consensus discussion (*n* = 28) and through a third reviewer (BP, 1 study referred). Of the 277 papers screened at full text by both reviewers, there was a 0.90 proportionate agreement probability, and a Cohen's Kappa of 0.68 showed good levels of agreement.

## 4 Inclusion and Exclusion Criteria

**FIGURE 1:**
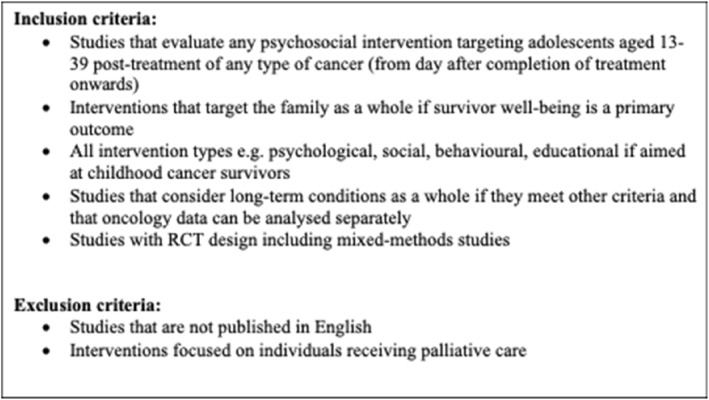
Inclusion and exclusion criteria.

**FIGURE 2:**
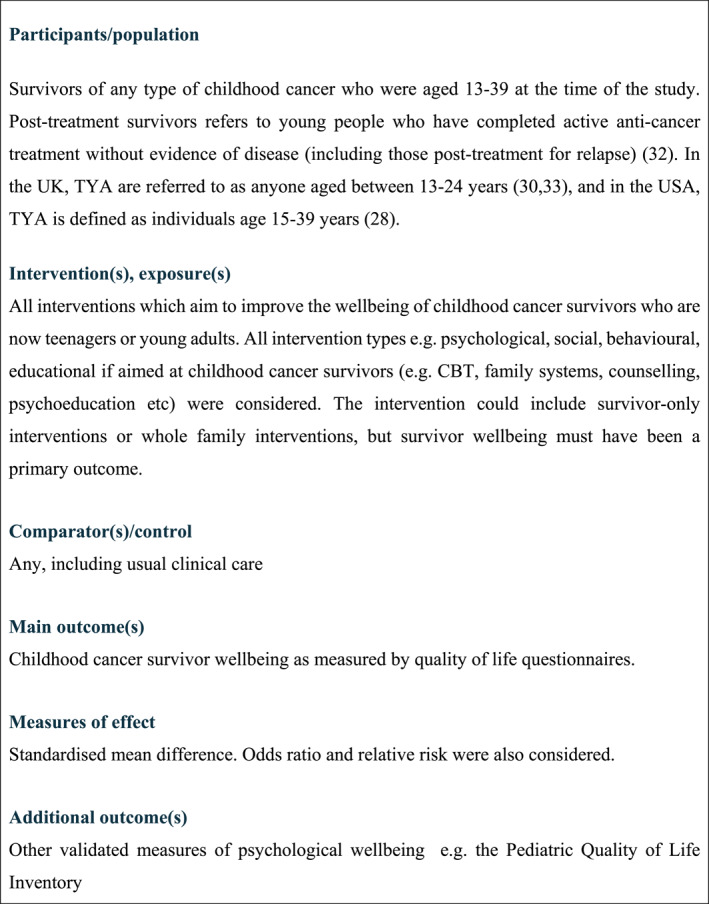
PICO criteria [31, 32].

Studies were included if they met the inclusion criteria shown in Figure 1 and PICO criteria in Figure 2.

### 4.1 Study Design

Only RCTs were included to minimise risk of bias [33].

## 5 Data Extraction and Quality Assessment (Risk of Bias)

**FIGURE 3:**
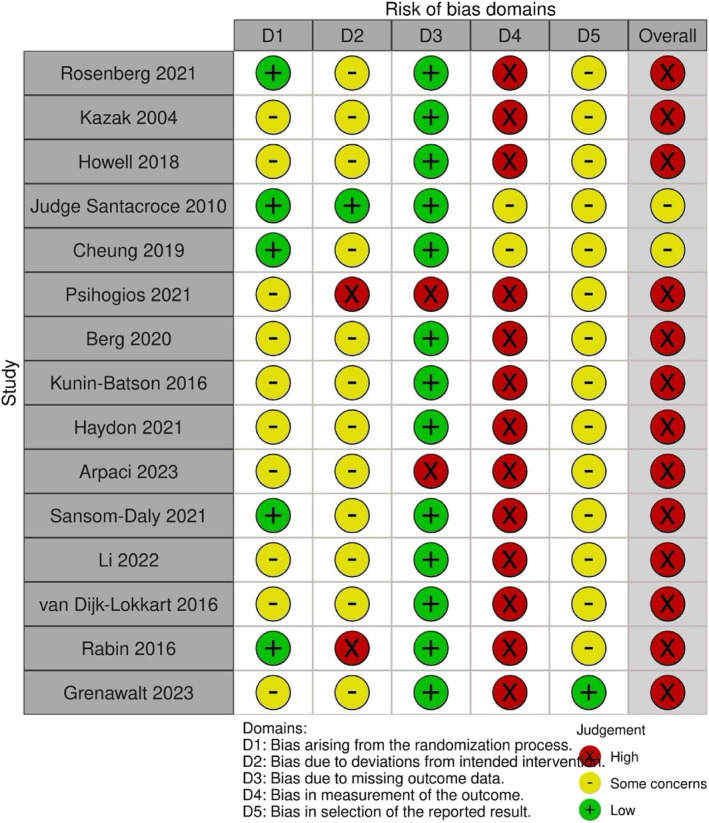
Risk of Bias assessment using RoB2.

Data was extracted by NOD and independently checked by LE. Risk of bias was assessed using version two of the Cochrane risk of bias for randomised trials (RoB 2). This tool provides a judgement of 'low risk' to 'high risk' about bias across five domains: randomisation process, deviations from intended interventions, missing outcome data, measurement of the outcome, and selection of the reported result. The assessment indicated variations in the risk levels across the assessed domains and studies. Figure 3 reports the judgements for each included study.

## 6 Results

### 6.1 Study Selection

**FIGURE 4:**
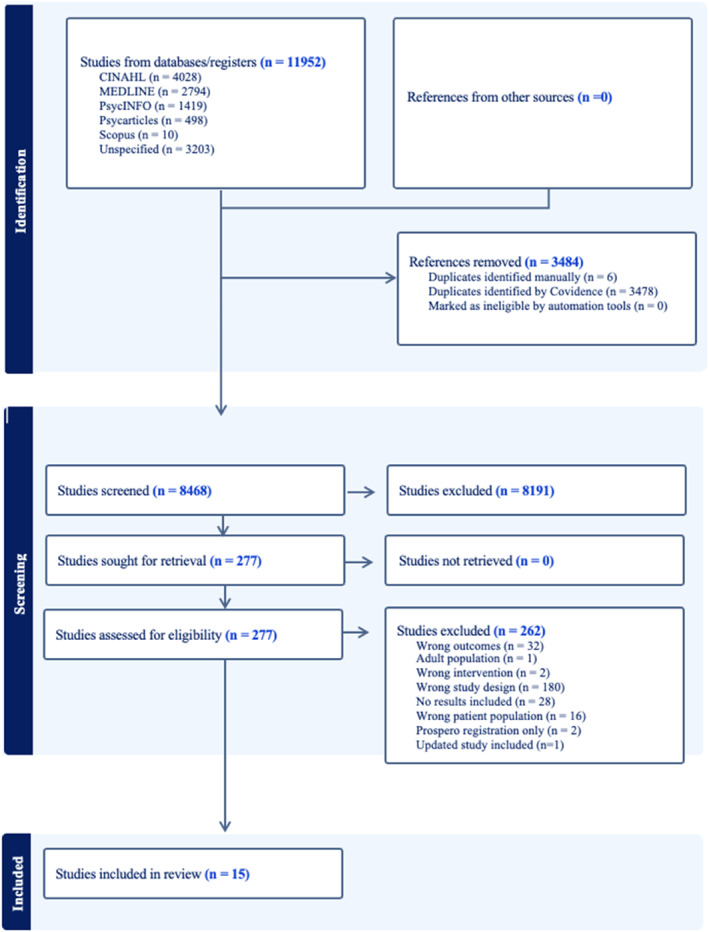
PRISMA flow diagram.

A total of 11,952 records were identified through the initial database search. After removing duplicates, 8468 articles remained for title and abstract screening. Following this screening, 277 articles were selected for full‐text review. Ultimately, 15 studies met inclusion criteria and were included in the final analysis. Of the 262 studies excluded at full text, some used study design other than RCT (*n* = 180), used outcome measures that were not validated measures of QoL or psychological well‐being (*n* = 32), was a protocol or did not include study results (*n* = 30), did not include TYA cancer survivors (*n* = 16), used an inappropriate intervention (*n* = 2), had an older adult population aged > 39 years (*n* = 1), or was an older version of an updated and included study (*n* = 1). See Figure 4.

### 6.2 Study Characteristics

Fifteen RCTs had sample sizes ranging from 21 to 253. Studies were published between 2004 and 2023, encompassing diverse populations and geographic locations; 10 in the United States of America (USA) [33, 34, 35, 36, 37, 38, 39, 40, 41] two in China [42, 43], one in Australia [26] one in Turkey [44], and one in the Netherlands [4]. See supplementary material for more detail.

## 7 Participants

A total of 1109 participants aged 8–39 years were included, with mixed populations of males and females included across studies. In 8 of 15 studies, more female participants were included than males, one study had a 50:50 gender split, and the remaining seven studies included more male than female participants. Participants were reported as survivors of many cancers, including: Blood (Leukaemia *n* = 161; Lymphoma *n* = 155), Brain and Central Nervous System (CNS) (Brain Tumour *n* = 148; CNS *n* = 24), Breast (*n* = 41), Bone (*n* = 22), Cervical (*n* = 2), Colorectal (*n* = 3), Germ Cell (*n* = 1), Melanoma (*n* = 10), Neuroblastoma (*n* = 6), Retinoblastoma (*n* = 11), Sarcoma (Soft Tissue Sarcoma *n* = 5; Ewing Sarcoma *n* = 1; Rhabdomyosarcoma *n* = 4), Solid Tumours (*n* = 115), Thyroid (*n* = 26), Testicular (*n* = 3), and other non‐specified (*n* = 134).

### 7.1 Interventions and Comparators

Interventions examined across studies included: an Education and counselling programme [45], the Achieving Wellness After Kancer (AWAKE) intervention [38], Musical training [46], FitSurvivor physical exercise training [4], Behavioural Activation (BA) intervention [39, 47], peer helping and expressive writing [40], web‐based physical activity intervention [39, 47], Craniosacral Therapy Technique (CTT) [48], The Surviving Cancer Competently Intervention (SCCIP) [42], Psychoeducation [43], Adolescent and Young Adult Self‐Management via Texting, Education, and Plans for Survivorship (AYA STEP) [44], RElaxation aNd Exercise for Wellness (RENEW) [48], Promoting Resilience in Stress Management (PRISM) [49] and the Recapture Life Intervention [30]. All 15 interventions were each evaluated in a single study that considered a different intervention. These were all modular and scheduled over multiple sessions, ranging from 4 to 52 weeks. They have been grouped into categories, highlighting similarities in delivery methods and area of focus (Table 1).

**TABLE 1:** Intervention categories.

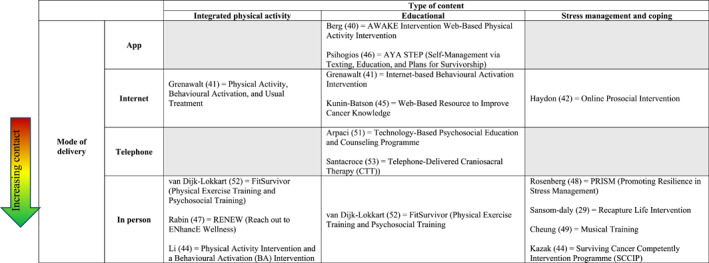

### 7.2 Data Synthesis and Statistical Analysis

A narrative synthesis was performed to provide an overview of main outcomes and trends. Meta‐analysis was not conducted because of the heterogeneity in interventions and outcomes reported. Although some outcomes were measured using the same tool across multiple studies, conducting meta‐analyses was deemed inappropriate for this review due to substantial variations in study design, intervention types, populations, and follow‐up periods. Even when, on occasion, the same outcome measures were used, differences in intervention duration, participant age range, and baseline characteristics introduced significant variability that could bias pooled effect estimates. The Cochrane guidelines [49] recommend meta‐analysis only when studies are sufficiently homogeneous in terms of key characteristics to ensure meaningful synthesis. Given the diversity observed, a narrative synthesis approach allowed us to more accurately interpret and describe the nuanced effects of interventions across diverse settings. This approach allowed us to observe which interventions showed promise in specific domains of mental well‐being, despite the variability among studies.

#### 7.2.1 Efficacy in Improving Survivors' Mental Well‐Being

Due to the wide variety of outcomes measured, the effectiveness of interventions is reported within the following categories of outcome; QoL, depression, anxiety, stress, mood, behaviour, self‐efficacy, coping, and support. For summary of findings see Table 2.

**TABLE 2:** Intervention influence on wellbeing and psychological health.

Outcome category	Effect direction	Author	Type of content			Mode of delivery				Findings
			Integrated physical activity	Educational	Stress management and coping	App	Internet	Telephone	In person	
QOL	Positive	Arpaci		✔				✔		Benefits at 12 months
		Cheung			✔				✔	
		Rosenberg			✔				✔	Benefits at 24 months
	Negative	van Dijk‐Lokkart	✔	✔					✔	Short‐term benefits in reducing pain and procedural anxiety Limited overall improvement
Depression	Positive	Berg		✔		✔				Potential efficacy
		Cheung			✔				✔	Reduced depressive symptoms in brain tumour survivors
		Grenawalt	✔	✔			✔			Improved life satisfaction and low mood symptoms in brain tumour survivors
		Haydon			✔		✔			Decreased depression over time from baseline to 1‐month follow‐up
	Negative	Sansom‐Daly			✔				✔	Peer support condition improved depressive symptoms Limited improvement in depressive symptoms
	No difference	van Dijk‐Lokkart	✔	✔					✔	No significant changes in depressive symptoms
Anxiety	Positive	Kazak			✔				✔	Marginal effect on fathers' anxiety
		Santacroce		✔				✔		Reduced anxiety in survivors; control group remained stable or deteriorated
	No difference	Haydon			✔		✔			No significant differences between intervention and control groups but decreased anxiety over time
		Kazak			✔				✔	No significant changes in anxiety for survivors, mothers, or siblings
		Kunin‐Batson		✔			✔			No significant differences between intervention and control groups on anxiety scales
Stress	Positive	Kazak			✔				✔	Reduced post‐traumatic stress symptoms
		Santacroce		✔				✔		Reduced stress and post‐traumatic stress in survivors and parents
		Rosenberg			✔				✔	Sustained improvements in stress and psychological distress
	No difference	Grenawalt	✔	✔				✔		No significant reduction in perceived stress
Mood	Positive	Rabin	✔				✔			Improved mood (POMS scale) associated with increased physical activity
		Haydon			✔				✔	Improved positive and negative affect over time
Behaviour	Positive	Santacroce		✔			✔			Improvements in behaviour, benefit finding, and health promotion
	No difference	van Dijk‐Lokkart	✔	✔				✔		No significant differences in behavioural challenges between intervention and control groups
Self‐Efficacy	Positive	Arpaci		✔					✔	Increased emotional self‐efficacy and coping scores at 3‐month follow‐up
		Li	✔					✔		Physical activity group showed sustained improvements at 1‐week and 3‐month follow‐ups compared to control and BA groups
Coping	Positive	Arpaci		✔					✔	Improved coping skills compared to routine follow‐up
		Sansom‐Daly			✔			✔		Increased use of adaptive coping strategies over time
Social Support	Positive	Li	✔						✔	Improved social support scores in the physical activity group
	No difference	Haydon			✔				✔	No significant improvements in social support over time

*Note:* **Positive** indicates improvement or beneficial outcome. **Negative** indicates limited improvement or other suboptimal outcomes. **No difference** indicates no statistically significant changes between intervention and control groups.

##### 7.2.1.1 QoL

Four studies utilised the Paediatric Quality of Life Inventory (The PedsQL 4.0) [46]; [4, 45, 46, 49]. The four different interventions were reported to have varying impact on survivor QoL. Three interventions were reported to show positive effects on QoL in the longer term (Arpaci's technology‐based psychosocial intervention *n* = 12 months, Cheung's musical training *n* = 12 months, Rosenberg's PRISM *n* = 24 months) whereas the other (van Dijk‐Lokkart et al.) indicated limited overall impact, with minor short‐term benefits [4]. van Dijk‐Lokkart et al. was the only intervention of the four to include physical exercise, and although they found short‐term positive parent‐reported effects on pain and procedural anxiety, there was found to be no significant overall improvement in QoL or well‐being.

##### 7.2.1.2 Depression

Six studies used depression measures, but none utilised the same scale; Berg [38] used the Patient Health Questionnaire‐9 item (PHQ‐9) [47], Cheung (2019) used the Centre for Epidemiological Studies Depression Scale for Children (CES‐DC) [45], van Dijk‐Lokkart et al. (2016) the Children's Depression Inventory [50], Grenawalt et al. (2023) used BA for Depression Scale—Short Form (BADS‐SF) [52], Haydon (2021) utilised the 20‐item Centre for Epidemiologic Studies Depression Scale [51], and Sansom‐Daly et al. (2021) used the Depression, Anxiety and Stress Scale‐Short Form depression and anxiety subscales [53].

Four interventions found improvements; the AWAKE app‐based intervention demonstrated potential efficacy, as did musical training which significantly reduced depressive symptoms in brain tumour survivors. Additionally, Grenawalt's internet‐based BA intervention positively impacted life satisfaction and symptoms of low mood in young adult brain tumour survivors. Haydon found a main effect of time on depressive symptoms in peer helping and expressive writing intervention groups, indicating decreased depression from baseline to 1‐month follow‐up. As with QoL, van Dijk‐Lokkart's intervention did not show significant changes in depressive symptoms post‐intervention between the intervention and control groups. Sansom‐Daly's online CBT programme found that participants across groups reported higher levels of depression at 12‐weeks and 12‐months post‐programme. This was in comparison to the peer support condition who showed improved symptoms.

##### 7.2.1.3 Anxiety

Three studies used anxiety measures, with Haydon [40] utilising the seven‐item generalized anxiety disorder scale (GAD‐7) [53], Kazak, Kunin‐Batson, and Santacroce [42, 43, 50] using the State–Trait Anxiety Inventory (STAI) [54], and Kazak [42] using the Revised Children's Manifest Anxiety Scale (RCMAS) [55].

Haydon's study of prosocial writing interventions demonstrated no significant differences in anxiety between intervention and control groups, though both decreased over time. Using the STAI, Kazak found that although the intervention had a marginal effect on fathers of survivors' anxiety, overall, it did not significantly impact anxiety levels in adolescent survivors, mothers, or siblings nor did it test this as an interaction. Santacroce's pilot study highlighted that the HEROS PLUS coping skills training (CST) intervention led to reduced anxiety in TYA survivors compared to those in the control group who remained the same or deteriorated. Kunin‐Batson did not find significant differences between intervention and control groups on anxiety scales.

##### 7.2.1.4 Stress

Four studies used measures of stress, with Kazak and Santacroce [42, 50] utilising the 20‐item Posttraumatic Stress Disorder Reaction Index [56], Rosenberg [49] using the Kessler‐6 psychological distress scale [57], and Grenawalt [39] utilising the Perceived Stress Scale (PSS‐10) [58].

Three of the four studies which measured stress outcomes found that their interventions had a positive impact. Kazak's study demonstrated the effectiveness of SCCIP, a CBT and family therapy approach, in reducing post‐traumatic stress symptoms. Similarly, Santacroce's HEROS PLUS CST intervention demonstrated reduced stress and post‐traumatic stress in both survivors and their parents. Rosenberg's PRISM intervention reported sustained improvements in stress and psychological distress. Grenawalt's BA intervention showed no significant reduction in perceived stress but positively impacted TYA's life satisfaction.

##### 7.2.1.5 Mood

Two studies collected data on mood, Rabin [48] using the Profile of Mood States (POMS) [59] and Haydon [40] using the Positive and Negative Affect Schedule (PANAS‐X) [60].

Rabin's study explored the effects of a physical activity and meditation intervention on mood. The intervention group showed significant improvement in mood as measured using the POMS scale. Analyses demonstrated significant interaction effects for the intervention group for both physical activity and mood improvement. Haydon used PANAS‐X to report improvements in both positive and negative affect over time. The study's adjusted means further highlighted a main effect of time, with symptoms decreasing from baseline to post‐intervention and baseline to 1‐month follow‐up.

##### 7.2.1.6 Behaviour

Two studies used two separate behavioural outcome measures, van Dijk‐Lokkart [4] used the Child Behaviour Checklist [61], and Santacroce [50] used the Health Promoting Lifestyle II [62]. The behaviours measured varied and could not be directly compared.

van Dijk‐Lokkart assessed behavioural difficulties (internalising: anxiety, depression, withdrawal; externalising: aggression, ‘delinquency’, hyperactivity) in survivors and while at baseline, a notable percentage of parents reported clinically significant total, internalising, and externalising challenging behaviours, there were no significant differences between the intervention and control groups after the intervention period. Santacroce, studying the HEROS PLUS intervention, focussed on coping skills training delivered by telephone, finding improvements in the behaviour of survivors and their parents.

##### 7.2.1.7 Self‐Efficacy

Two studies used two separate measures of self‐efficacy, Arpaci [45] used the Self‐Efficacy Questionnaire for Children (SEQ‐C) [63], and Li [47], the General Self‐efficacy Scale (GSES) [64]. Arpaci's technology‐based intervention showed a significant increase in emotional self‐efficacy scores for the intervention group over time. Li found that the physical activity group, but not BA group, of their intervention demonstrated a significant difference in self‐efficacy in comparison to the control group at 3 months post‐intervention.

##### 7.2.1.8 Coping

Two studies examined coping outcomes, both Sansom‐Daly and Santacroce [30, 45] using the KIDCOPE [65].

Sansom‐Daly's study focused on coping strategies in two different interventions: Recapture Life (CBT) and a peer‐support group. Participants in both interventions reported increased use of coping strategies 6‐week post‐intervention, but with survivors in the CBT group demonstrating more adaptive coping strategies at 12 weeks than those in the peer‐support group. Arpaci's intervention was also found to significantly improve coping skills of survivors compared to the routine long‐term follow‐up control group.

##### 7.2.1.9 Social Support

Two studies assessed support outcomes, both Haydon and Li [40, 47] using the 21‐item 2‐way Social Support Scale (2‐Way SSS) [66].

Haydon's study focussed on prosocial interventions, specifically peer helping and expressive writing with peer helping, evaluating effects on social support, but did not demonstrate significant improvement over time. Li's study investigated the impact of physical activity on social support. The physical activity intervention group demonstrated statistically significant, higher scores compared to the behaviour activity group and the control group at both 1‐week and 3‐months post‐intervention.

##### 7.2.1.10 Miscellaneous

Eleven studies utilised 23 different outcome measures that did not fall into any other category, and are reported in the notes.

#### 7.2.2 Psychosocial Interventions and Positive Influence on Well‐Being and Psychological Health of TYA Survivors

Three interventions were found to positively influence QoL: Rosenberg, Cheung, and Arpaci [45, 46, 49]. Additionally, van Dijk‐Lokkart's physical exercise intervention showed minor short‐term benefits but had limited overall impact on QoL. In terms of depression, several interventions showed improvements [38, 39, 40, 46]. For anxiety, Haydon's prosocial interventions, particularly peer helping and expressive writing with peer helping, showed benefit in participants experiencing increased wellbeing and greater social support compared to the control group. Kazak's SCCIP intervention, Santacroce's HEROS PLUS telephone‐based craniosacral intervention, and Rosenberg's PRISM intervention positively impacted stress and post‐traumatic stress symptoms. Rabin's physical activity and meditation intervention and Haydon's peer helping and expressive writing interventions showed improvements in mood. Santacroce's HEROS PLUS CST intervention positively impacted health promotion behaviour and benefit finding in survivors and their parents. Both Arpaci's technology‐based intervention and Li's physical activity intervention demonstrated sustained positive impact on self‐efficacy. Sansom‐Daly's Recapture Life CBT programme and Arpaci's psychosocial education intervention had positive results in coping skills. Additionally, Li's physical activity intervention found a positive impact on social support.

#### 7.2.3 Psychosocial Interventions and Negative Impacts or ‘Adverse Events’ on Well‐Being and Psychological Health of TYA Survivors

van Dijk‐Lokkart's intervention and Sansom‐Daly's Recapture Life online CBT programme were not found to positively influence QoL. In addition, Kazak's SCCIP did not significantly impact anxiety levels in survivors, mothers, or siblings. Grenawalt's BA intervention showed no significant reduction in perceived stress, and again, van Dijk‐Lokkart's intervention showed no significant differences in behavioural challenges between intervention and control groups. Despite not having positive effects, none of the included interventions were reported to have adverse effects on well‐being and/or psychological health.

## 8 Discussion

The synthesis of the included studies provides a comprehensive overview of the diverse available psychosocial interventions aiming to improve psychosocial well‐being of TYA cancer survivors. Despite this, the heterogeneity in outcome measures and intervention types poses many challenges in drawing definitive conclusions. Future research should strive to use standardised outcome measures and consistent sample sizes, compared by diagnosis and age of cancer experience, to enhance comparability and allow for meta‐analyses. Additionally, follow‐up studies are needed to assess the sustainability of intervention effects across multiple studies and long‐term. This should also include interventions being tested in more than one trial to ascertain reproducibility. The lack of long‐term follow‐up data in the current review makes it difficult to draw conclusions about any lasting benefits of interventions. This could be rectified through testing across multiple time points and through the use of longitudinal studies. The absence of explicit reporting on adverse effects also emphasises the importance of systematically evaluating and reporting both positive and negative outcomes in future research. This absence highlights a broader issue within psychosocial intervention studies, where reporting of both positive and potential negative outcomes remains inconsistent. A standardised framework for documenting adverse effects alongside beneficial outcomes would strengthen future reviews by providing a more balanced perspective on intervention efficacy and safety. Incorporating such guidelines could enhance the comparability and rigour of future studies, offering a more comprehensive understanding of intervention impacts on well‐being.

In terms of limitations of the review itself, the reported evidence on psychosocial interventions for TYA survivors is derived exclusively from RCTs. Although RCT is considered the ‘gold standard’ in assessing the efficacy of therapies [67], those utilising different experimental designs are not captured, and thus some meaningful and effective interventions may be overlooked. Furthermore, the inclusion of studies only published in set countries could potentially have impacted conclusions. Equally, the inclusion of studies only written in the English language means that this may not be representative to survivors in non‐English speaking countries or with different cultures to the origins of the included studies. Excluding these studies reduces the comprehensiveness of the review and may result in a narrower understanding of their efficacy and applicability across diverse populations. Lastly, a limitation in assessing whether psychosocial interventions had higher efficacy was the diversity of primary outcome measures, including QoL, anxiety, and depression. This variability meant that differences in observed efficacy may partly reflect the nature of the outcomes themselves, such as differences in the sensitivity of measures to detect change, the initial levels of participants, and the specific construct assessed. Additionally, as meta‐analyses were not conducted due to heterogeneity in outcomes and intervention types, efficacy was assessed descriptively, limiting the ability to make definitive comparisons. Standardised outcome measures in future research would enhance the feasibility of robust statistical comparisons of efficacy across studies.

### 8.1 QoL

Interventions targeting QoL had mixed effects. While Cheung's music training, Rosenberg's PRISM stress management, and Arpaci's technology‐based psychosocial education programme showed positive impact, van Dijk‐Lokkart's physical exercise intervention showed limited overall improvement. As with all of the included studies, variability in intervention types and outcomes emphasises the necessity for a nuanced understanding of the factors influencing TYA survivors' QoL. Cheung's music training, for instance, might have positively affected TYA survivors by providing an outlet for emotional expression and fostering a sense of community. Rosenberg's intervention could have addressed specific stressors associated with cancer survivorship, offering coping mechanisms and support. Arpaci's technology‐based psychosocial education programme may have targeted information gaps and provided valuable resources for survivors. On the other hand, the limited success of van Dijk‐Lokkart's physical exercise intervention prompts considerations about the appropriateness of certain interventions for this demographic. The constraints could stem from physical limitations, preferences, or motivational factors unique to TYA survivors. The variability in intervention types and outcomes across studies signals the heterogeneity within the TYA survivor population. Factors such as age, cancer type, treatment history, and individual preferences can significantly impact effectiveness of interventions. Therefore, a one‐size‐fits‐all approach is unlikely to yield consistent results, highlighting the need for personalised and flexible strategies.

### 8.2 Depression

Studies which measured depression utilised completely different outcomes, making direct comparisons challenging. However, interventions such as Berg's AWAKE app, Cheung's musical training, Grenawalt's internet‐based BA, and Haydon's peer helping and expressive writing interventions showed potential in lessening symptoms of depression. This highlights the potential of different interventions in addressing depressive symptoms, where present, in survivors. However, the challenge of comparing studies due to the utilisation of different outcomes to measure depression complicates the synthesis of evidence and the identification of overarching trends. The lack of standardised measures may introduce variability and limit the ability to draw clear conclusions about the effectiveness of these interventions. In addition, comparing single studies of different interventions does not allow for nuanced understanding of impact. Use of standardised assessments for depression could enable more robust comparisons and facilitating clearer understanding of the effectiveness of different interventions.

### 8.3 Anxiety

Studies investigating anxiety also had nuanced results. Haydon's prosocial interventions exhibited trends towards increased social support and reduced symptoms of anxiety. Santacroce's HEROS PLUS CST intervention also had positive effects. In contrast, Kazak's family focussed intervention showed no significant impact on survivors, mothers, or siblings despite having a moderate impact on fathers' anxiety. The mixed findings across studies could emphasise the heterogeneity across included studies. As with all factors, anxiety may differ based on individual characteristics, family dynamics, and coping mechanisms. As such, a generalised approach to anxiety management may be ineffective. Understanding the nuances of family interactions and their differential impact on anxiety levels among survivors is essential for tailoring interventions to achieve optimal outcomes.

### 8.4 Stress

Interventions targeting stress outcomes demonstrated varying efficacy. Kazak's SCCIP intervention, Santacroce's HEROS PLUS CST, and Rosenberg's PRISM reported positive impacts on stress and post‐traumatic stress symptoms. However, Grenawalt's BA intervention showed no significant reduction in perceived stress. Despite this, comparators of results and outcomes across disparate measures and interventions are invalid. The discrepancy in outcomes may refer to the need for greater understanding of specific mechanisms through which interventions impact stress. It is also plausible that these findings may have been due to an underpowered study or the use of imprecise reporting measures. However, results suggest the need for targeted stress‐reduction strategies in interventions for this population.

### 8.5 Mood

Studies focusing on mood outcomes generally indicated positive effects, particularly with Rabin's physical activity and meditation intervention and Haydon's peer helping and expressive writing interventions. These findings underscore the potential of physical and psychosocial interventions to improve mood among TYA survivors. Yet it is important to note potential limitations and consider broader implications. The positive effects observed could be influenced by various factors such as study design, sample size, and participant characteristics. Replicating these findings in diverse populations and contexts would enhance the generalisability of these interventions. Secondly, the specific mechanisms through which physical and psychosocial interventions impact mood outcomes need further exploration. Understanding the underlying processes can guide the development of more targeted interventions, as well as help in adapting them to individual preferences and needs.

### 8.6 Behaviour

Behavioural outcomes were only assessed in two studies, with mixed results. While van Dijk‐Lokkart's physical exercise intervention showed no significant differences in behavioural difficulties, Santacroce's HEROS PLUS CST positively impacted health promotion behaviours. Future studies should place greater emphasis on evaluating behavioural outcomes among survivors. This includes not only assessing the presence of behavioural difficulties but also exploring the promotion of positive health‐related behaviours. The multifaceted nature of behavioural changes suggests that interventions should be tailored to address specific aspects of behaviour, acknowledging the specific challenges faced by TYA survivors.

### 8.7 Self‐Efficacy

Both Arpaci's technology‐based intervention and Li's physical activity intervention demonstrated positive impacts on self‐efficacy, emphasising that interventions focussing on enhancing TYA's beliefs in their emotional and coping abilities may be beneficial across different populations. However, the long‐term sustainability of the observed positive impacts on self‐efficacy is a critical consideration. It is essential to assess whether these improvements endure over time and whether they contribute to lasting positive changes, given the decades of life TYA survivors have to manage the impact of their experiences.

### 8.8 Coping

Studies focussing on improving coping skills, including Sansom‐Daly's Recapture Life CBT programme and Arpaci's psychosocial education intervention, highlighted the benefit of different strategies on improving coping skills, suggesting that this is an important element of support tools for TYA childhood cancer survivors. However, it is crucial to delve deeper into specific components of these interventions that contribute to improved coping skills, and test whether these benefits exist outside the single study. Understanding the active ingredients and mechanisms of change can guide the development of more targeted and effective coping interventions.

### 8.9 Social Support

Interventions targeting social support, such as Haydon's prosocial intervention and Li's physical activity intervention, showed positive impacts. Strengthening social support networks appeared to be a key component in enhancing overall well‐being. It is important that the sustainability of the observed positive impacts on social support is considered. Evaluating whether the strengthened social networks endure over time and contribute to ongoing well‐being is essential for assessing the long‐term effectiveness of these interventions. The implications of these findings highlight the integral role of social support in enhancing overall well‐being among survivors. Future research should continue to explore and refine strategies that effectively foster social support, recognising the diverse needs within the TYA survivor community and promoting adaptability for sustained positive outcomes.

### 8.10 Study Limitations

The included studies share some common methodological limitations. An issue across several studies was small sample size. For instance, Arpaci (*n* = 62) and Berg (*n* = 56) both faced challenges associated with limited statistical power and the ability to detect significant differences. This highlights the need for interventions to be tested with larger, more diverse samples to ensure findings are robust and clinically meaningful.

Bias, in terms of participant selection and the methods used, was evident in several studies. Grenawalt's study primarily consisting of White, educated males, and the use of a convenience sample strategy introduced further potential biases. Equally, the inclusion of predominantly American literature is not fully transferable to other settings. Additionally, the lack of literature from the UK may reflect the lack of RCT funding for psychosocial interventions. The overrepresentation of one specific demographic may inadvertently perpetuate existing disparities in cancer care and overlook the experiences and perspectives of those from underrepresented backgrounds, who may be in greater need of psychosocial support. Kazak's study demonstrated issues with high dropout rates and biases associated with home‐based data collection, compromising the internal validity of findings and introducing confounding variables that may have influenced the findings. Santacroce's intervention relied on telephone‐delivery, which may have led to biases related to participant preferences and needs.

Lastly, high dropout rates were a recurring challenge, although statistical methods were used to account for these. Kazak acknowledged the differential dropout rates between the intervention and wait‐list control groups, with higher rates in the intervention group. This could suggest that there may have been specific challenges or barriers that affected acceptability of the intervention for some individuals. Similarly, Sansom‐Daly's research had an underpowered sample and high attrition rates, impacting the representativeness of the results and suggesting difficulties in maintaining participant commitment throughout the study duration. This may indicate potential issues with acceptability of the intervention or study design, such as the demands of multi‐session interventions, or burden of multiple questionnaires over a 12‐month period. These consistent issues with retention highlight the importance of addressing factors contributing to attrition and devising strategies to enhance participant adherence in future research.

### 8.11 Clinical and Research Implications

Various interventions demonstrated positive influence on different aspects of well‐being among TYA childhood cancer survivors. Prosocial interventions, physical activity, and technology‐based psychosocial education programmes exhibited the most consistent positive effects across multiple domains. Therefore, such interventions in oncology settings could promote social connections and emotional wellbeing amongst TYA survivors. However, conducting more in‐depth research on these aspects in the future is needed, before implementation. Establishing guidelines for professionals to adopt such approaches when designing interventions, as well as integrating comprehensive survivorship psychosocial care plans alongside medical interventions to provide holistic support at all stages, is also needed. Ultimately, tailored, and multifaceted approaches considering the individual needs of this group of cancer survivors are crucial for optimising intervention efficacy. It is also important for clinicians to be clear about which interventions are effective, and if there is a choice of empirically tested support, providing guidance on which might best suit their patient group. These, however, must be rigorously tested and monitored for bias to ensure reliable findings across individual interventions.

## 9 Conclusions

This review highlights the potential of psychosocial interventions positively impacting the mental well‐being of TYA childhood cancer survivors, contributing valuable insights to the ongoing efforts to enhance the QoL and psychological health of this population. This is increasingly crucial as more TYA survive their diagnoses and must live with potential late effects that can vastly impact psychological well‐being. Despite this, comparing single studies of differing interventions does not provide full understanding of nuanced differences and is subject to reporting bias. Concerted effort is needed to improve understanding of which elements of interventions are helpful, neutral, or detrimental. This may include combining data from different study designs, conducting multiple studies on the same intervention, as well as considering diverse cancer diagnoses and populations of TYA. It is hoped that this review highlights existing support and acts as a guide for considering the development of future interventions.

## Author Contributions

**Nicola O'Donnell:** conceptualisation, data curation, methodology, resources, formal analysis, project administration, writing–original draft, writing–review & editing. **Leila Ellis:** data curation, formal analysis, writing–review & editing. **Jessica E. Morgan:** supervision, writing–review & editing. **Debra Howell:** supervision, writing–review & editing. **Pernille Axél Gregersen:** resources, writing–review & editing. **Victoria Willard:** conceptualisation, resources, writing–review & editing. **Bob Phillips:** conceptualisation, funding acquisition, supervision, writing–review & editing.

## Ethics Statement

This review contains data derived from published sources only.

## Conflicts of Interest

The authors declare no conflicts of interest.

## Data Availability

Data are reported fully in the included tables and figures and are taken from published and publicly available sources only.
